# In dogs with atopic skin disease, is lokivetmab more effective than oclacitinib in reducing the score of a recognised scoring system?

**DOI:** 10.18849/ve.v7i2.569

**Published:** 2022-06-22

**Authors:** Bonnie Yuan Tone Cheung+

**Keywords:** ATOPIC DERMATITIS, CANINE, IL-31, IMMUNOMODULATORY THERAPY, JAK-KINASE SIGNLALLING, LOKIVETMAB, OCLACITINIB, PRURITUS

## Abstract

PICO question

In dogs with atopic skin disease, is lokivetmab more effective than oclacitinib in reducing the Canine Atopic Dermatitis Lesion Index score (or some other recognised scoring system)?

Clinical bottom line

Category of research question

Treatment

The number and type of study designs reviewed

One randomised controlled trial and one before and after study were critically appraised

Strength of evidence

Weak

Outcomes reported

One randomised controlled trial studied the effects of lokivetmab and oclacitinib and found that both drugs were similar in reducing the Canine Atopic Dermatitis Lesion Index (CADESI-03) score.

An additional study was evaluated but had non-standardised data as it was a before-and-after study on use of lokivetmab. The paper noted that dogs’ response to oclacitinib can be used to predict how well these dogs respond to lokivetmab. This study also reported a reduction in Pruritus Visual Analog Scale (PVAS) score between before and after lokivetmab administration

Conclusion

In view of the strength of evidence and outcomes from the studies, there is insufficient quality of evidence to answer the PICO question and so further comparative study is required

How to apply this evidence in practice

The application of evidence into practice should take into account multiple factors, not limited to: individual clinical expertise, patient’s circumstances and owners’ values, country, location or clinic where you work, the individual case in front of you, the availability of therapies and resources.

Knowledge Summaries are a resource to help reinforce or inform decision making. They do not override the responsibility or judgement of the practitioner to do what is best for the animal in their care.

## Clinical scenario

You are a small animal veterinarian working in a private practice where one of your clients brings in a dog with a history of licking and itching parts of their body. Upon clinical examination, you find the axillae, feet, face, and groin are severely pruritic with no secondary bacterial infection present. The clients note that the dog is already on a hypoallergenic diet, has flea and worming treatment up to date, and is currently on glucocorticoids which owner notices is not as effective as it was before in pruritic control. Therefore, you wish to use immune-modulating drugs: oclacitinib or lokivetmab as they are relatively new to the market and have been approved for use in dogs with canine atopic dermatitis (CAD). The client has no restrictions financially, but wants to get their beloved pet’s itching and pruritus under control. Oclacitinib is administered orally and given initially twice a day for 2 weeks and then once a day whereas lokivetmab is given as a subcutaneous injection every 4 weeks. Oclacitinib and lokivetmab target similar pathways known to reduce pruritus, e.g., IL-31, so when choosing, which one is better?

## The evidence

There were many studies investigating the efficacy of oclacitinib and lokivetmab individually in dogs diagnosed with canine atopic dermatitis (CAD) (Cosgrove et al*.*, 2013; Fleck et al*.*, 2015; Michels et al*.*, 2016(a); Michels et al*.*, 2016(b); Marsella & Ahrens, 2018; Szczepanik et al*.*, 2019; and Van Brussel et al., 2021), but few compare the effects of both drugs in the same study. To investigate efficacy of drugs for CAD, scoring systems have been useful to provide a comparable method of assessment. A common scoring system used is Canine Atopic Dermatitis Extent and Severity Index (CADESI), which is utilised by clinicians for its thorough assessment of CAD patients. There are currently four iterations, the latest being CADESI-04 (Olivry et al*.*, 2014). Each iteration assesses the patient using a different list of body areas to score severity of lesions (e.g., erythema, lichenification). Overall scores between iterations cannot be compared. Therefore, extrapolating scores from different studies that individually studied the efficacy of oclacitinib and lokivetmab cannot be used reliably for this Knowledge Summary.

However, there are other scoring systems like Pruritus Visual Analog Scale (PVAS), which is more accessible for owners to use when assessing CAD (Hill et al., 2007), or cosmetic assessment by owners from Bensignor & Videmont (2021) but this has not currently been validated. Moreover, overall PVAS scores from studies which have assessed either efficacy of oclacitinib or lokivetmab cannot be used for this Knowledge Summary as these are subjective to owners’ perception and bias. Therefore, PVAS scores from different studies cannot be used for direct comparison to assess whether lokivetmab is better than oclacitinib. For this Knowledge Summary, only CADESI and PVAS scoring systems were used to compare the efficacy of oclacitinib and lokivetmab because these have been used more widely across studies and have been validated in assessing severity of CAD (Hill et al., 2007; Olivry et al*.*, 2007; and Olivry et al*.*, 2014).

Therefore, two studies were found to be relevant to the PICO question, but there was only one controlled study directly comparing the effects between lokivetmab and oclacitinib using CADESI-03 and PVAS (Marsella et al., 2020). An additional study was evaluated because it included some comparison between lokivetmab and oclacitinib treatment using PVAS, but this was a before-and-after study where oclacitinib was assessed before lokivetmab treatment (Souza et al*.*, 2018).

## Summary of the evidence

**Marsella et al. (2020):** 

Population:	Atopic Beagle dogs: 8 years old. Intact females (n = 9), intact males (n = 9) and neutered male (n = 1). Maintained in a research facility (University of Florida).
Sample size:	19 dogs.
Intervention details:	All dogs were fed the same diet, housed where runs were cleaned daily and there were no toys that trap dust. All dogs were at a research facility sensitised to *Dermatophagoides farinae *and were exposed to the allergen twice a week. Dogs were housed in pairs, but it is unclear how they were allocated. A pair of dogs were allocated to receive either: Oclacitinib – Oral administration of 0.5 mg/kg twice daily for 2 weeks then once daily for 2 weeks. Ciclosporin – Oral administration of 5 mg/kg once a day for 28 days. Lokivetmab – Subcutaneous administration of 2 mg/kg on first day of allergen challenge. Prednisone – Oral administration of 5 mg/kg twice daily for 2 weeks, then 0.5 mg/kg once daily for 1 week and then 0.5 mg/kg once every 48 hours for 1 week. Control – No treatment. Allocation of dogs in each group was not mentioned.
Study design:	Randomised, controlled, blinded study.
Outcome studied:	Canine Atopic Dermatitis and Extent Severity Index, third iteration (CADESI-03) scoring: Subjective assessment by investigator who was unaware of treatment allocation. Performed once on Day 0, twice on the days of the allergen challenge (before exposure and 6 hours after), and 24 hours after each allergen challenge. Allergen challenges were on days 2, 6, 9, 13, 16, 20, 23, and 27. Behavioural Observation Research Interactive Software (BORIS) recording: Subjective assessment performed by two people who were unaware of treatment allocation. This was used to score the duration(s) of pruritic behaviour (e.g., licking, biting and scratching). When the dog was biting or scratching, the observer pressed a key which indicated the start of behaviour and then pressed again to indicate the end. For licking behaviour, each lick was indicated by a key press. Camera recordings were taken on Day 0 and on days 2, 9, 16 and, 23, recordings were taken 4 hours after allergen exposure. Pruritus Visual Analog Scale (PVAS): Subjective assessment performed by the same evaluators that reviewed the BORIS recordings. Scoring scale from 0 to 10 where 0 was described as ‘no itching is observed’ whereas 10 was described as ‘severe itching, manifested as interruption of eating, playing or resting, in order to itch’. Transepidermal water loss (TEWL): Objective assessment performed on days 0, 14 and 28. Measured as evaporation rate (g/m^2^/h) using a closed chamber device (VapoMeter®) at ambient temperature (20–26°C). Three unclipped areas were evaluated (concave surface of pinnae, axilla and inguinal). Hydration: Objective assessment performed on days 0, 14 and 28. Measured using a Corneometer® where a probe is placed perpendicular to the skin for 10 seconds and expressed as micro-Siemens (μS). For statistical analysis, the four weeks of study was divided into two periods (T1/Acute phase = first 2 weeks, T2/Chronic phase = final 2 weeks). Statistical evaluation of data was performed using mixed-model restricted maximum-likelihood 5-group x 2-time Analysis of Variance (REML-ANOVA) with a between-subjects factor of group (control, ciclosporin, lokivetmab, oclacitinib, prednisone) and within-subject factor of time (T1, T2). Area under the curve (AUC) was calculated for each dog (CADESI-03 scores, TEWL and hydration for each site examined). Additionally, Kruskal-Wallis test R was used to determine differences between groups whereas Friedman’s test was used to investigate the effect of time. Significance was set at P < 0.05.
Main Findings (relevant to PICO question)	Oclacitinib had a statistically significantly lower CADESI-03 score than controls during T1 (P = 0.015) whereas there was no statistical significance found for the lokivetmab-treated group. At day 28, dogs treated with lokivetmab and oclacitinib had mild to no erythema from pictures of a representative dog in each group. No statistical significance was detected for mean PVAS as well as AUC for the duration of scratching between T1 and T2 phase for either lokivetmab or oclacitinib treatment. On day 28, controls had a higher TEWL than the lokivetmab-treated group (P = 0.031) in pinnae and axillary regions, but none reported for the oclacitinib-treated group. No changes in TEWL were seen in oclacitinib- and lokivetmab-treated groups for any of the assessed regions over time. AUC for hydration over time were higher in lokivetmab- and oclacitinib-treated groups (P = 0.014 and P = 0.04, respectively) which authors presume that dogs in these groups were more hydrated than control dogs. CADESI-03 and TEWL show positive correlation (r = 0.21, P = 0.0043) which authors suggest is a positive effect for oclacitinib and lokivetmab treated groups as there is no further inflammation due to CAD.
Limitations:	Even though authors mention the study being randomised, there is no detail in the methodology section about how this was carried out and how many dogs were assigned in total to each treatment or control group. Small sample size: there was no information about the power of the study and why sample size of 19 dogs was chosen. And so, conclusions of no statistical significance will need to be interpreted with caution. Limited body areas measured for TEWL but this limitation has been acknowledged by the authors. Subjective assessment by investigators using CADESI-03 and PVAS but has been used consistently for assessing recordings as well as scoring the dogs in the study. CADESI-03 and PVAS are both validated scales but CADESI-03 has undergone an amendment to become CADESI-04 which is simpler, quicker to administer and more selective for body sites where there is high sensitivity and specificity for CAD diagnosis (Olivry et al*.*, 2014). There was very little presentation of data obtained in terms of BORIS recordings in the results section, especially since time of pruritic behaviour was included in statistical analysis. Pruritic reactions were experimentally induced, hence are not representative of allergens which typically cause CAD in a clinical context.

**Souza et al. (2018):** 

Population:	Medical records at Colorado State University Veterinary Teaching Hospital from November 2015 to the end of October 2016. Dogs were treated for allergic dermatitis and received lokivetmab. Diagnosis of atopic dermatitis (AD) was made if pruritus was seasonal (considered showing signs of persistent allergic dermatitis for more than 1 year) or by exclusion if dog failed to have an adverse food reaction (AFR) (positive response to an 8 week restrictive diet using either novel protein or hydrolysed diet). Dogs were further diagnosed into three main groups: AD, AFR and AD, allergic dermatitis of undetermined cause (ADUC), based on case history and clinical signs as well as ruling out other pruritic diseases through appropriate diagnostic tests and / or therapeutic trials. Medications for treatment of allergic dermatitis were either discontinued prior to lokivetmab injection or maintained at their current doses and frequencies of administration.
Sample size:	135 dogs.
Intervention details:	Age of dogs and dose of lokivetmab (mg/kg) at initial administration was recorded. Frequency of dosing ranged between every 2–7 weeks. Dogs included in study were treated with one or more lokivetmab injection. AD (n = 80; seasonal = 7 and non-seasonal = 73), AFR and AD (n = 10) and ADUC (n = 45).
Study design:	Retrospective, before and after study.
Outcome studied:	Subjective assessment by owners: Owners documented severity of pruritus at each visit with a Pruritus Visual Analog Scale (PVAS) score. PVAS consists of a 10 cm line with descriptions at 2 cm intervals. All individuals were sequentially grouped based on severity of pruritus: Very severe (>8–10) Severe (>6–8) Moderate (>4–6) Mild (>2–4) Very mild (>0–2) Treatment success was empirically defined as ≥2 cm reduction in PVAS score from baseline on the day of assessment. More than 50% reduction of PVAS score compared to baseline on day of assessment day was also recorded. Time to clinical improvement after initial lokivetmab administration: Assessed by owners either within 24 hours, between 1 and 3 days or after 3 days. Frequency of administration required to maintain control of pruritus was recorded (weeks). Number of dogs initially classified as treatment success but failed to respond to subsequent lokivetmab injections was noted in addition to the number of injections prior to encountering this lack of response. Previous therapy for CAD: Dose, frequency of administration and success (no response, partial response or total response) were recorded based on previous records or client’s report. Dogs that had failed oclacitinib but responded to lokivetmab were recorded. Previous responses to ciclosporin, corticosteroids and immunotherapy were recorded in categories: no response, partial response or total response. Concurrent therapies: Any dog receiving another treatment for allergic dermatitis / pruritus at time of initial lokivetmab administration was recorded (e.g. allergen-specific immunotherapy), including the duration prior to lokivetmab administration. Concurrent illnesses: Dogs that had concurrent allergic conditions and systemic and / or cutaneous diseases were noted, including their response to lokivetmab treatment. Adverse events: Documentation of adverse events associated with lokivetmab administration or any of the various therapies used concurrently with lokivetmab. Statistical analysis: Treatment success was defined as ≥2 cm reduction in PVAS score. Categorical data was expressed as frequencies, whereas continuous data was expressed as median and range. Wilcoxon matched pairs signed-rank test was used to analyse PVAS scores reported after drug administration compared to pretreatment scores. Factors that were potentially associated with response included: type of allergic disease duration of clinical signs, age and size of dog, lokivetmab dosage, previous response to oclacitinib or other therapy (ciclosporin, corticosteroids, immunotherapy, dietary trial), concurrent therapy with a previous response and pruritus severity prior to lokivetmab. These factors were evaluated for association with two outcomes: dichotomous outcome of treatment success or failure, and magnitude of change, measured by difference in PVAS score after and prior to treatment. Univariate logistic regression, yielding odds ratio (OR) and 95% confidence intervals (CI) and linear univariate regressions were used to associate continuous and categorical risk factors with outcome variables. Pearson’s chi-squared test was used to evaluate dichotomous risk factors for treatment success or failure when cell counts were greater than 5 whereas two-tailed Fisher’s exact test was used when cell counts were 5 or less. Significance was set at P < 0.05.
Main Findings (relevant to PICO question)	Response to lokivetmab treatment: Prior to lokivetmab administration, 135 dogs had a median PVAS score of 6.5 (range 1–10), but after lokivetmab administration, 132 dogs had a median PVAS score of 1.5 (range 0–8.5). This decrease in PVAS score pre- and post-lokivetmab administration was found to be statistically significant (P <0.001). There was no outcome information for 3/135 dogs. Improvement of pruritus in 116/132 (87.8%) of dogs following lokivetmab administration at 1.8–3.7 mg/kg. Oclacitinib therapy as predictor of lokivetmab success: 66/135 dogs had received oclacitinib for pruritus control prior to lokivetmab and were treated for at least 3 weeks. 45/66 dogs had total response to oclacitinib when receiving it twice daily whereas the other 21 dogs had partial or no response to twice daily oclacitinib. 40/45 dogs that had total response to twice daily oclacitinib were partially responsive to once daily oclacitinib. 37/40 dogs were lokivetmab responders. 15/21 dogs that had partial or no response to twice daily oclacitinib were also lokivetmab responders. Odds of lokivetmab treatment success on average was 7.4 times higher (95% CI = 1.3–40.9; P = 0.03) for dogs showing total response to twice daily oclacitinib (and partial response to once daily dosing) when compared to dogs with incomplete response to twice daily oclacitinib therapy.
Limitations:	Not a controlled study. Non-standardised PVAS between clinicians (i.e., not the same clinician assessing all the dogs in the study) hence this introduces a bias in giving scores. No supporting pruritus assessment (e.g., CADESI-04) performed by clinician. Subjective assessment using PVAS by owners as well as assessing time to improvement which could introduce bias to the data and placebo-effect. Inclusion of other concurrent treatments and illnesses, e.g., glucocorticoids which could overestimate the effects of lokivetmab treatment. PVAS assessment time-frame not standardised as it was performed with each visit to the hospital. Frequency of lokivetmab injections were not the same for every dog included in the study which affects the data assessing efficacy of lokivetmab over a fixed period of time. Unclear about meaning of no / partial / total response when assessing other therapies for pruritus.

## Appraisal, application and reflection

Canine atopic dermatitis (CAD) is one of the most common skin diseases in general practice which is diagnosed through the elimination of other pruritic skin disorders (e.g., flea allergic dermatitis, sarcoptic mange or cutaneous adverse food reaction) as well as history (seasonal or non-seasonal). CAD is defined as ‘a genetically predisposed inflammatory and pruritic allergic skin disease with characteristic clinical features associated with immunoglobulin E (IgE) antibodies most commonly directed against environmental allergens’ (Halliwell, 2006). The disease is often non-specific in cause, but it is a lifelong condition which requires constant monitoring and pruritus control.

Typically, treatment for CAD includes glucocorticoids (e.g., prednisolone), topical steroids and ciclosporin which are drugs that have sufficient evidence for efficacy (Olivry et al., 2002). However, adverse effects can occur associated with long-term use of glucocorticoid treatment, or gastrointestinal signs (e.g., vomiting) in the case of ciclosporin (Steffan et al., 2003). Topical therapy tends to be reserved for short-term and / or localised therapy but commonly in CAD, many dogs present with more than one region of pruritus and / or greater in severity. Therefore, newer treatments are immune-modulating drugs such as oclacitinib and lokivetmab which selectively inhibits Janus kinase-1 receptor and IL-31, respectively.

Overall, there was relatively little evidence available directly comparing the effects of lokivetmab and oclacitinib in studies using an updated scoring system, e.g., CADESI-04, consistently. Hence, extrapolating from other studies which individually assessed the efficacy of either oclacitinib or lokivetmab with a placebo cannot be used due to the different scoring systems utilised. For instance, CADESI-03 and CADESI-04 focus on different areas of the body to determine overall score and severity of pruritus and so the scores cannot be used to justify whether lokivetmab has more efficacy than oclacitinib (Olivry et al*.*, 2007; and Olivry et al*.*, 2014). Additionally, scoring systems such as CADESI and PVAS are subjective to the clinician or owner and so comparing the scores between studies cannot reliably determine whether lokivetmab has more efficacy than oclacitinib in treating CAD.

Marsella et al. (2020) reported that lokivetmab is more effective for preventing flare ups given before an allergy challenge, but oclacitinib and lokivetmab overall had similar effects on transepidermal water loss (TEWL) and hydration. Despite the paper mentioning it was randomised, there were no details as to how this was carried out in the trial. Therefore, the lack of randomisation and small sample size limits the value of the study which performing a sample size calculation before recruiting dogs for the study would have strengthened the data collected. Additionally, authors reporting no statistical significances between mean PVAS scores could be influenced by small number of dogs in study and so results should be interpreted with caution.

In Souza et al. (2018), it is a retrospective before-and-after study which collected data that was already recorded, but since it is neither a randomised nor controlled trial, there are many variable factors included in the study. For example, the inclusion of dogs with concurrent systemic and / or cutaneous illnesses as well as other concurrent treatments (e.g., corticosteroids) adds another limitation to the data because these could influence the outcomes detected and overestimate the effectiveness of lokivetmab. Furthermore, the frequency of lokivetmab treatment varied from 2–7 weeks which authors argued that lokivetmab treatment was given as frequent as needed to keep pruritus under control. However, this does not truly assess the effectiveness of lokivetmab over a fixed period of time and so limits the strength of the data. Moreover, many studies using lokivetmab typically administered every 4 weeks which is also recommended by Cytopoint® dosage chart (AH430/17) (Moyaert et al., 2017; and Szczepanik et al., 2020). Additionally, increasing lokivetmab frequency to more than every 4 weeks shows a decline in efficacy in terms of PVAS scores (Michels et al., 2016[a]). Hence, in the Souza et al. (2018) study, there are many confounding factors which have not been accounted for.

Since the PVAS assessment in Souza et al*. *(2018) were only performed by owners, there is certain bias to the data collected e.g., time to improvement after initial lokivetmab administration, as well as introducing a placebo-effect. The authors also suggest that previous response to oclacitinib is a good predictor for response to lokivetmab therapy – dogs that responded poorly to oclacitinib respond well to lokivetmab therapy. However, knowing that oclacitinib is a Janus kinase-1 inhibitor whereas lokivetmab is a monoclonal antibody to IL-31, it is understandable that blocking IL-31 which is further upstream will inhibit other downstream pathways including the Janus kinase-1 pathway. Since CAD is a multi-faceted disease, it is recommended that a combination of treatments should be used for optimal benefit (Olivry et al., 2015).

The evidence above is insufficient to say one drug is more effective than the other, but both treatments are certainly effective in the treatment for CAD and potentially are safe to use together (Gortel, 2018). Since there are various scoring systems used in the assessment of CAD, only PVAS has been consistently used across the literature whereas CADESI has undergone several iterations and so is not as easily comparable for efficacy of oclacitinib and lokivetmab. Therefore, comparing PVAS scores between placebo-controlled trials for either oclacitinib or lokivetmab suggest both treatments result in similar outcomes (average score of 2–3 from treatment up to day 28 after administration) (Cosgrove et al., 2013; Michels et al., 2016[a]; Szczepanik et al., 2020; and Van Brussel et al*.*, 2021). This, however, is a very crude comparison and what is inferred cannot compare to randomised controlled trials (RCT) directly observing and assessing the efficacy of oclacitinib and lokivetmab together.

Furthermore, there has not been any further updated guidelines from the International Committee on Allergic Diseases of Animals (ICADA) since 2015 which does not include recommendations for lokivetmab, but for oclacitinib, its short-term use is shown to be beneficial in treating CAD (Olivry et al*.*, 2015). As a result, there is simply not enough evidence available to answer the PICO question and further randomised controlled studies are required to demonstrate the efficacy of both lokivetmab to oclacitinib.

## Methodology Section

**Search Strategy:** 

Databases searched and dates covered:	CAB Abstracts on OVID Platform (1973–Week 48 2021) PubMed on the NCBI interface (1920–December 2021)
Search strategy:	CAB Abstracts: (dog or dogs or canine or canines or bitch or bitches).mp. or exp dogs/ or exp canis/ or exp bitches/ (atopic and (skin or pruritus or pruritis or dermatitis)).mp. (Lokivetmab or Cytopoint or Oclacitinib or Apoquel).mp. 1 and 2 and 3 PubMed: dog OR bitch OR canine atopic and (skin or pruritus or pruritis or dermatitis) Lokivetmab or Cytopoint or Oclacitinib or Apoquel 1 and 2 and 3
Dates searches performed:	7 Dec 2021

**Exclusion / Inclusion Criteria:** 

Exclusion:	Non-English language study, conference papers, case studies, review articles and articles that did not compare lokivetmab and oclacitinib in atopic dogs and so was not relevant to PICO question.
Inclusion:	Peer-reviewed, controlled studies comparing the effects between lokivetmab and oclacitinib in dogs with atopic dermatitis using a recognised scoring system.

**Search Outcome:** 

Database	Number of results	Excluded – Non-English language publication	Excluded – Not relevant to PICO question	Excluded – Case studies in English	Excluded – Conference papers	Excluded – Reviews in English	Total relevant papers
CAB Abstracts	76	22	26	6	8	12	2
PubMed	39	0	32	1	0	4	2
Total relevant papers when duplicates removed							2

## Conflict of interest

The authors declare no conflicts of interest.

The author would like to thank Dr Kit Sturgess for his guidance with this article and Clare Boulton for her assistance in obtaining papers.

## Disclaimer

Knowledge Summaries are a peer-reviewed article type which aims to answer a clinical question based on the best available current evidence. It does not override the responsibility of the practitioner. Informed decisions should be made by considering such factors as individual clinical expertise and judgement along with patient’s circumstances and owners’ values. Knowledge Summaries are a resource to help inform and any opinions expressed within the Knowledge Summaries are the author's own and do not necessarily reflect the view of the RCVS Knowledge. Authors are responsible for the accuracy of the content. While the Editor and Publisher believe that all content herein are in accord with current recommendations and practice at the time of publication, they accept no legal responsibility for any errors or omissions, and make no warranty, express or implied, with respect to material contained within. For further information please refer to our Terms of Use.
